# The miR-125a and miR-320c are potential tumor suppressor microRNAs epigenetically silenced by the polycomb repressive complex 2 in multiple myeloma

**DOI:** 10.14800/rd.1529

**Published:** 2017-04-03

**Authors:** Mohammad Alzrigat, Helena Jernberg-Wiklund

**Affiliations:** Science for Life Laboratory, Department of Immunology, Genetics and Pathology, Rudbeck Laboratory, Uppsala University, Uppsala, SE-751 85, Sweden

**Keywords:** Multiple Myeloma, PRC2, EZH2, H3K27me3, microRNA

## Abstract

We have previously presented the histone methyltransferase enhancer of zeste homolog 2 (EZH2) of the polycomb repressive complex 2 (PRC2) as a potential therapeutic target in Multiple Myeloma (MM). In a recent article in Oncotarget by Alzrigat. *et al*. 2017, we have reported on the novel finding that EZH2 inhibition using the highly selective inhibitor of EZH2 enzymatic activity, UNC1999, reactivated the expression of microRNA genes previously reported to be underexpressed in MM. Among these, we have identified miR-125a-3p and miR-320c as potential tumor suppressor microRNAs as they were predicted to target MM-associated oncogenes; IRF-4, XBP-1 and BLIMP-1. We also found EZH2 inhibition to reactivate the expression of miR-494, a previously reported regulator of the c-MYC oncogene. In addition, we could report that EZH2 inhibition downregulated the expression of a few well described oncogenic microRNAs in MM. The data from our recent article are here highlighted as it shed a new light onto the oncogenic function of the PRC2 in MM. These data further strengthen the notion that the PRC2 complex may be of potential therapeutic interest.

Multiple myeloma (MM) is a malignancy of plasmablasts/plasma cells (PCs) characterized by the accumulation of monoclonal antibody producing PCs in the bone marrow (BM). Clinically, MM is a heterogeneous disease and MM patients’ show multiple clinical symptoms including lytic bone lesions, anemia, hypercalcemia, renal failure and immunodeficiency ^[1–3]^. More importantly, MM is a biologically complex disorder characterized by a large clonal heterogeneity as reflected by a wide range of genetic alterations and manifested in a patient-to-patient variation in overall survival and response to treatment ^[4–7]^. This MM associated heterogeneity has certainly limited the clinical benefits of current as well as personalized treatment strategies. Therefore, MM remains a fatal disease making development of new targeted therapeutic approaches imperative.

Several reports from genetic sequencing and gene expression studies in MM have documented a cross-talk between genetic lesions and aberrant epigenetic profiles i.e. DNA methylation ^[8, 9]^, histone modifications ^[10, 11]^ and non-coding RNA ^[12–14]^ in the pathogenesis and prognosis of MM. An emerging notion is now that deregulation of epigenetic modifiers is an important factor contributing to the development of MM ^[15–18]^. For example, the chromosomal translocation t(4;14) results in the overexpression of the multiple myeloma set domain (MMSET) histone methyltransferase leading to an increase in histone 3 lysine 36 di-methylation (H3K36me2) levels and a concomitant decrease in histone 3 lysine 27 tri-methylation (H3K27me3) levels ^[10, 11]^. The enhancer of zeste homolog 2 (EZH2) is an epigenetic modifier that has been shown by us and others to be commonly overexpressed in MM ^[16, 19, 20]^. EZH2 is the enzymatic subunit of the polycomb repressive complex 2 (PRC2), an important regulator of both normal development as well as disease ^[21–23]^. Through EZH2, the PRC2 complex establishes the H3K27me3 mark, a transcriptional repressive histone mark involved in the regulation of transcriptional programs during normal development as well as cellular transformation ^[21–23]^. EZH2 was found to be overexpressed in malignant PCs as compared to normal BM PCs, and to enhance MM cell growth ^[19]^. Recently, we have shown that a common set of PRC2/H3K27me3 targeted genes are underexpressed in MM patients ^[20, 24]^. Stressing the clinical relevance of gene silencing by PRC2, we found that the repression of PRC2 target genes (H3K27me3 targets) in MM correlates with gene silencing in advanced stages of MM and in patients presenting with poor survival ^[24]^. The development of epigenetic inhibitors that specifically dampen the EZH2 enzymatic activity has recently made the evaluation of the therapeutic potential of EZH2 in MM possible. We and others have demonstrated the anti-MM effects mediated by EZH2 inhibition by using highly selective inhibitors of the EZH2 enzymatic activity ^[24–26]^. All these studies reported on the anti-MM effects of EZH2 inhibitors via reactivation of a set of PRC2 target genes with anti-tumor functions such as genes involved in apoptosis, cell differentiation, cell adhesion and migration.

As here highlighted, we have recently reported for the first time that inhibition of EZH2 using the small highly selective inhibitor of EZH2 enzymatic activity, the UNC1999 ^[27]^, has an impact on the global expression of microRNA genes in MM. In this study we presented PRC2 as a novel regulator of a set of microRNAs with tumor suppressor or oncogenic function in MM ^[28]^. In the study, we found that EZH2 inhibition by UNC1999 resulted in the upregulation of 118 microRNAs, of which many have been identified as downregulated tumor suppressor microRNAs in MM ^[28]^. We could show that 2 potential tumor suppressor microRNAs, miR-125a-3p and miR-320c, were reactivated upon EZH2 inhibition (Figure 1). We selected these microRNAs based on their predicted binding and function as common regulators of MM important oncogenes i.e. IRF-4, XBP-1 and BLIMP-1 ^[28]^. We also found that UNC1999 upregulated the expression of miR-494 with a previously reported function to negatively regulate the expression of the c-MYC oncogene ^[29]^. Using chromatin immunoprecipitation followed by quantitative real time PCR (ChIP-qPCR), we found that miR-125a and miR-320c were direct targets of PRC2 in MM cell lines and primary MM patient cells and that their reactivation, as predicted, correlated with the downregulation of expression of MM-associated oncogenes IRF-4, XBP-1, BLIMP-1 and c-MYC ^[28]^. The significance of our finding relies on the fact that these oncogenes have been demonstrated to be essential for MM cell growth and survival ^[30–33]^ and MM pathogenesis in human and murine models ^[34–37]^.

We also showed that the inhibition of EZH2 downregulated the expression of microRNAs reported to be overexpressed and to possess oncogenic functions in MM ^[28]^. Among these, the miR-17-92 and miR-106b-25 clusters in MM have been attributed oncogenic functions due to their regulation of MM associated tumor suppressor genes. For example, members of the miR-17-92 cluster have previously been shown to target the tumor suppressors SOCS1 and BIM ^[38, 39]^. Similarly miR-106b-25 in MM has been suggested to modulate the activity of the tumor suppressor P53 ^[38, 40]^. Furthermore, members of the miR-17-92 cluster and Let-7 family were suggested to enhance MM angiogenesis ^[41]^, an important step in MM establishment and progression. Interestingly, the expression of miR-17-92 and miR-106b-25 clusters is positively modulated by c-MYC in tumors other than MM ^[42, 43]^. Therefore, we suggest that EZH2 inhibition in MM may indirectly affect the expression of onco-miRNAs via downregulation of MM-associated oncogenes such as c-MYC.

Recent analysis of microRNA expression in MM revealed that deregulation of microRNA expression correlates with molecular subtype, disease progression, patients’ survival and response to treatment ^[13, 14, 44–47]^. Several reports have suggested genetic lesions such as chromosomal translocations and copy number variations ^[13, 14, 46, 47]^, but also epigenetic mechanisms e.g. DNA methylation ^[48, 49]^ as possible mechanisms leading to aberrant expression of microRNA genes in MM. For example, DNA methylation was shown to epigenetically silence the expression of tumor suppressor microRNAs such as miR-155, miR-198, miR-135a*, miR-200c, miR-663 and miR-483-5p ^[50]^. Our recent findings expand the knowledge concerning the regulation of microRNA expression in MM and suggest also polycomb-mediated gene repression as a mechanism that may deregulate and silence tumor suppressor microRNAs. Overexpression of tumor suppressor microRNAs or their mimics have in some cases been proven to have anti-MM activity inhibiting MM cell growth, migration and colony formation *in vitro* ^[48, 50]^ and *in vivo* ^[51, 52].^ The possibility of using microRNAs as a novel therapeutic strategy in MM should thus be the subject for further investigation.

In summary, our recent publication ^[28]^ demonstrated for the first time PRC2 as a regulator of microRNA expression in MM, thus emphasizing the oncogenic role of EZH2 in MM. Our present findings show that EZH2 inhibition leads to upregulation of a set of tumor suppressor microRNAs targeting important MM-associated oncogenes, and suggest EZH2 inhibitors and the silenced tumor suppressor microRNAs as possible novel therapeutic strategies in MM.

## Figures and Tables

**Figure 1:**
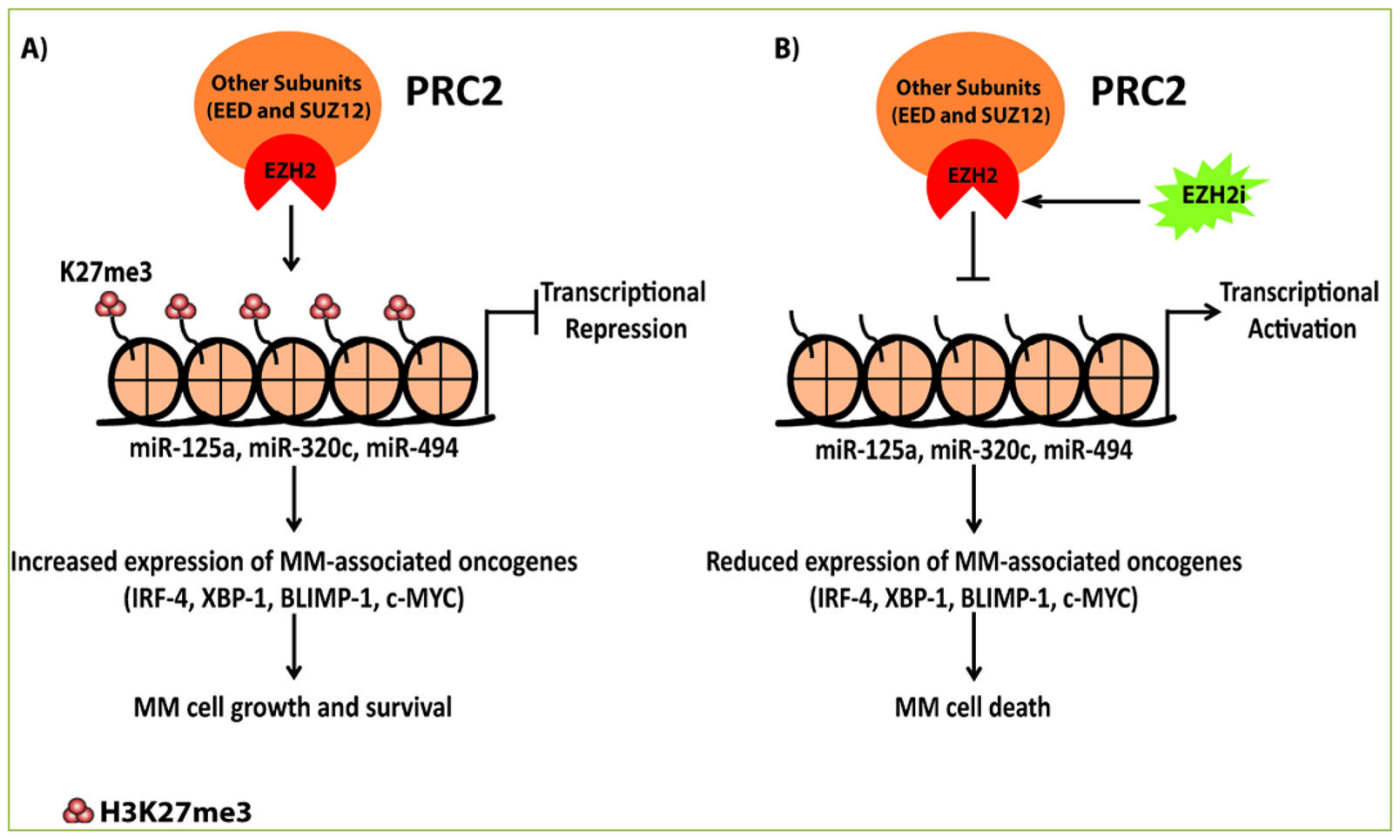
PRC2 represses the expression of tumor suppressor microRNAs in multiple myeloma (a) PRC2 via EZH2 enzymatic subunit installs H3K27me3 mark at the genes encoding miR-125a, miR-320c and miR-494 in MM cells leading to their silencing. This leads to sustained expression of MM-associated oncogenes predicted to be targets of the repressed microRNA resulting in enhanced MM cell growth and survival. (b) Pharmacological inhibition of EZH2 methyltransferase activity using highly selective inhibitors such as UNC1999 abolishes the installation of H3K27me3 at the microRNA genes, leading to their transcriptional activation. Expression of miR-125a, miR-320c and miR-494 leads to reduced expression of MM-associated oncogenes thus inducing MM cell death.

## Abbreviations

BM: bone marrow
EZH2: enhancer of zeste homolog 2
H3K27me3: histone 3 lysine 27 tri-methylation
H3K36me2: histone 3 lysine 36 di-methylation
miR: microRNA
MM: multiple myeloma
MMSET: multiple myeloma set domain
PCs: plasma cells
PRC2: polycomb repressive complex 2
